# Relationships of *Helicoverpa armigera*, *Ostrinia nubilalis* and *Fusarium verticillioides* on MON 810 Maize

**DOI:** 10.3390/insects2010001

**Published:** 2011-01-20

**Authors:** Béla Darvas, Hajnalka Bánáti, Eszter Takács, Éva Lauber, Árpád Szécsi, András Székács

**Affiliations:** Plant Protection Institute, Hungarian Academy of Sciences, H-1022 Budapest, Herman Ottó u. 15., Hungary; E-Mails: banati@julia-nki.hu (H.B.); etakacs@julia-nki.hu (E.T.); eva.lauber@gmail.com (É.L.); aszecsi@nki.hu (Á.Sz.); aszek@nki.hu (A.Sz.)

**Keywords:** *Ostrinia nubilalis*, *Helicoverpa armigera*, *Fusarium verticillioides*, MON 810, yield loss

## Abstract

MON 810 maize was developed against *Ostrinia nubilalis* and is suggested to indirectly decrease *Fusarium* spp. infestation in maize ears. To evaluate this effect, co-occurrence of insect and fungal pests on MON 810 maize was studied. During 2009, exceptionally high maize ear infestation occurred in Julianna-major (Hungary). From investigation of some thousands of maize ears, the majority of the larval damage originated from *Helicoverpa armigera* larvae, while *O. nubilalis* larvae contributed significant damage only at a single plot. *Fusarium verticillioides* infection appeared only in a small portion (∼20–30%) of the insect damaged cobs. *H. armigera* and *O. nubilalis* larvae feeding on *F. verticillioides* mycelia can distribute its conidia with their fecal pellets. MON 810 maize showed 100% efficacy against *O. nubilalis* in the stem, but lower efficacy against *O. nubilalis* and *H. armigera* in maize ears. The ∼Cry1Ab toxin content of maize silk, the entry site of *H. armigera*, was lower than that in the leaves/stem/husk leaves of MON 810. *Fusarium*-infected MON 810 cobs are rarely found and only after larval damage by *O. nubilalis. H. armigera* larvae could not tolerate well *F. verticillioides* infected food and attempted to move out from the infected cobs. For further feeding they re-entered the maize ears through the 8–12 husk leaves, but in the case of the MON 810 variety, they usually could not reach the kernels. Apical damage on cobs resulted in only a minor (about one-tenth of the cob) decrease in yield.

## 1. Introduction

MON 810 maize, expressing a transgene encoding a truncated version of the lepidopteran-specific Cry1Ab toxin of *Bacillus thuringiensis* var. *kurstaki* [1], was developed against *Ostrinia nubilalis* (Hübner) (Lep., Crambidae) and is suggested to indirectly decrease *Fusarium* spp. infestation [2,3] resulting in decreased mycotoxin content in the genetically modified (GM) crop [3–5]. Different additional lepidopteran pests of maize occur in the American continents and in Europe, *i.e. Helicoverpa zea* (Boddie) (Lep., Noctuidae) in North America, *Spodoptera frugiperda* (J. E. Smith) (Lep., Noctuidae) in South America, *Helicoverpa armigera* (Hübner) (Lep., Noctuidae) in Eastern Europe and *Sesamia nonagrioides* (Lefebvre) (Lep., Noctuidae) in Western Europe [3,6]. *O. nubilalis* is a stem and maize ear pest, while larvae of European noctuid species damage only maize ears. In the Pannonian Region, where maize ear damage mostly originates from *O. nubilalis* and *H. armigera*, strong infestation by *O. nubilalis* occurs only about once every ten years [7]. In the ongoing experimental cultivation series at Julianna-major (the Ecological Research Station, Plant Protection Institute), where maize has been cultivated in several plots during the last decade, significant infection caused by *H. armigera* and *O. nubilalis* larvae occurred only in 2009. Presently, after soil-disinfection pesticides (formerly used against Melolonthidae and Elateridae larvae) have been withdrawn, Hungarian farmers do not use chemical insecticides in maize production. The low frequency of maize ear damage caused by lepidopterous larvae may question the economic usefulness of MON 810 maize in this region [7]. Presently, the owner of the genetic event offer MON 810 maize varieties with the rational that lower larval damage of maize ears may reduce *Fusarium* spp. infestation, therefore, fusariotoxin contamination of such kernels is lower. A certain professional debate exists in this field: some groups claim that maize ear rot is associated with feeding damage caused by *O. nubilalis* and *H. zea* larvae in the U.S. [3], others found this association apparent in the case of *O. nubilalis* only [4]. The aim of this study was to clarify such pest co-occurrence in Hungary, one of the greatest maize producers in Europe.

## 2. Experimental Section

### 2.1. Stock Colonies

Potato dextrose agar was used for stock culture of *F. verticillioides* originating from a single microconidium (storage: 10 °C) [8]. Laboratory stock colonies of *O. nubilalis* and *H. armigera* were established. For a stock colony of *O. nubilalis*, adults were collected during 2004 in Kéty (Hungary). Larvae were fed on a semi-synthetic diet [9]. For breeding of *H. armigera*, larvae were collected during 2008 in Zsámbék (Hungary). Larvae were fed on a different semi-synthetic diet [10].

### 2.2. Laboratory Work

In a preliminary examination, fecal pellets of *H. armigera* were collected in a maize field (Páty, August 8, 2009). The fecal samples contained *F. verticillioides* (∼80%) and *F. proliferatum* (∼20%) microconidia, thus further experiments were focused on *F. verticillioides.* RbGUM selective media was used for *Fusarium* species isolation (incubation: 25 °C, 7–10 days). Insect and plant samples under field conditions were collected and examined using RbGUM media [8].

Lepidopteran larvae reared for the field experiments in maize ear isolators were fed on sweet maize (Jubilee) discs with or without *F. verticillioides* mycelia. To prepare a *F. verticillioides* infected disc, sweet maize cobs were cut to 3 cm thick discs. Discs were dipped into an aqueous solution (200 mL) including a stock culture of *F. verticillioides* and 0.1% Tween 20. The final concentration was ∼80,000 microconidia/mm^3^. After four days (incubation: 22–25 °C), when the distribution of mycelia was at least ∼50% on the surface of a single disc, second or third instar larvae were transferred onto them. Larvae were fed on sweet maize discs with or without *F. verticillioides* mycelia until the next molt.

### 2.3. Work in Maize Ear Isolators

Field work was done at the Ecological Research Station, Plant Protection Institute at Julianna-major (the north-western outskirts of Budapest). Maize ear isolators were placed on MON 810 or near isogenic plants at the beginning of silking time (August 13, 2009). Isolators were placed on artificially infected cobs using third or fourth larval stadia. The stem was cut above the maize ear at the height of maize silk, an appropriately treated larva was placed on it, and the fine mesh maize ear isolator (diameter = 10 cm, length = 35 cm) was placed on the entire maize ear and was tightly closed at the bottom. Treatments were: (i) *H. armigera* larvae having consumed *F. verticillioides*; (ii) *H. armigera* larvae not having consumed *F. verticillioides*; (iii) *O. nubilalis* larvae having consumed *F. verticillioides*; (iv) *O. nubilalis* larvae not having consumed *F. verticillioides* mycelia; and (v) untreated control (Table 1). Repetitions were 10–14 for *H. armigera* and 18–20 for *O. nubilalis*. A treated control (vi; 20 repetitions) consisting of *F. verticillioides* mycelia on RbGUM media disc (5 mm diameter) fixed on maize silk was also used. After two weeks, maize ear isolators were collected and their content was carefully checked.

### 2.4. Measurement of Cry1Ab Toxin Content in Maize Ear Parts of MON 810

Cry1Ab toxin content in the main parts of the maize ears was determined by a commercial sandwich immunoassay, Abraxis Bt-Cry1Ab/Ac ELISA kit (#PN 51001, Warminster, PA, USA) carried out in 96-well microplates according to manufacturer-provided protocol. ELISA signals were detected on an iEMS microtiter plate reader (Labsystems, Helsinki, Finland). Cry1Ab protoxin calibrators (Abraxis) were put on every microplate at concentrations between 0.25 and 4 ng/mL, assays were used for determination of analyte concentration by linear regression. Activated Cry1Ab toxin concentrations were calculated from detected protoxin concentration values with Cry1Ab activated toxin/protoxin cross-reactivity, 56.4%, previously determined for the Abraxis Bt-Cry1Ab/Ac ELISA kit [11,12].

### 2.5. Work under Field Conditions

A MON 810 (DK-440 BTY) and its near isogenic line (DK-440) were investigated during October 5–8, 2009, in Julianna-major. Depending on the plot size, some hundreds (*ca.* 100–400) of maize ears were collected from every assortment. Upon removal of husk leaves, cobs were carefully investigated for symptoms and severity of larval and fungal damage (proportion of the damaged part in the cob, location of the damage, occurrence of pink ear rot as a sign of fungal infection). Four replicates were used resulting in 457 to 1,339 cobs investigated per a single plot. Data without transformation were analyzed using Statistica ver. 5.5 program (ANOVA and Tukey test). Insect and plant samples were also collected for *Fusarium* spp. identification in the laboratory.

To evaluate the real yield loss, cobs (10 replicates) in different sizes (small, medium and big size of cob) infected by *H. armigera* larva were chosen. The infected part of the cobs was removed and masses were measured.

## 3. Results

### 3.1. *Fusarium verticillioides* Transmission Caused by Individually Isolated Larvae

*Fusarium verticillioides* was identified in laboratory fungal rearing tests (using *Fusarium*-selective RbGUM media) from plant and fecal pellet samples collected by the maize cob isolators in the field experiments. Thus, *F. verticillioides* infection was verified in fecal pellets of *H. armigera* and *O. nubilalis*, husk leaves, ripening kernels and cobs from maize plants damaged by *H. armigera* and *O. nubilalis*, as well as stem tunnels caused by *O. nubilalis*. Insect-transmitted infestation of *F. verticillioides* (manifested in the occurrence of pink ear rot) appeared to be dependent on the choice of the plant part by the insect to feed on, and on the varying subsequent survival rates due to different Cry1Ab toxin exposures. In contrast, no infestation by *F. verticillioides* was observed in the untreated control, and none was produced by fixing *F. verticillioides* mycelia on corn silk, either (Table 1). All the *H. armigera* larvae used (third and fourth stadia) chose maize ears for feeding. The majority of *O. nubilalis* larvae (third and fourth stadia) preferred to feed on the fresh wound on the stem, where it had been cut prior to the placement of the maize ear isolators on isogenic maize. A small proportion (10–15%) of *O. nubilalis* larvae tended to feed on husk leaves on MON 810 maize (Table 1). Larvae of both species investigated, having been fed previously in laboratory rearing during one larval instar on *F. verticilliodes* mycelia, showed increased mortality (10–17%). *F. verticillioides* infection was transmitted only by larvae having been fed previously on its mycelia (Table 1). Nonetheless, not all larvae (*H. armigera* and *O. nubilalis*) having been fed on *F. verticillioides* mycelia could transmit *Fusarium* infestation; the rate was only 20–42%. All of the *O. nubilalis* larvae died on MON 810 maize, although only this species could transmit *F. verticillioides* infection feeding on the base of maize ears. Some *H. armigera* larvae (third and fourth stadia) could survive on MON 810 maize, feeding on husk leaves (Table 1). These larvae stop feeding from time to time, and starve—showing the symptoms of Cry1-toxicosis—consuming the minimum possible.

### 3.2. *Helicoverpa armigera, Ostrinia nubilalis* and *Fusarium verticillioides* Infection under Field Conditions

Similarly high levels of maize ear infestation (42–46%) were observed in all three, closely located maize fields investigated. Only 20–30% of cobs with larval damage were also infected by *F. verticillioides*. Larval damages were mostly attributed to *H. armigera* in two of the maize fields, while both *H. armigera* and *O. nubilalis* larvae occurred in nearly identical rates in the third case (Table 2). Independently from the damaging insect species, mostly apical cob infestation occurred. *Fusarium verticillioides* infestation was significantly higher in one case, but it did not correlate with the overall larval damages (Table 2).

In case of *O. nubilalis*, the tunnels were found mostly on stems, and only a quarter of the damage occurred on maize ears (Table 3).

The position where the larvae fed on the maize ears were different for the two species evaluated. In the case of *H. armigera*, ∼90% of the damage occurred on the apical region. A lower value (∼80%) of apical damage was related to *O. nubilalis* (Table 4). First instar larvae of both species usually try to reach the kernels through the maize silks, although a more significant portion of *O. nubilalis* larvae choose the base of maize ear. In case of microbial infection (like *F. verticillioides*), older *H. armigera* larvae might move (∼10%) toward the middle of the cob, seeking a drier environment, or come out from the maize ear at the top and choose another maize ear. In this latter case, older *H. armigera* larvae choose husk leaves to reach the middle of the cob. The most frequent damage type by *H. armigera* larvae occurs on the top of the cob by feeding on ripening seeds. An ample amount of fecal pellets may be found at places of earlier seeds. Fecal pellets of *H. armigera* are sometimes covered by mycelia of different fungi. In our case, plant pathogenic *F. verticillioides* was the most abundant (Table 5). The damage type caused by *O. nubilalis* larva is different. It makes a longer tunnel toward the base of the cob under the seed surface. Fecal pellets of *O. nubilalis* are usually not visible on the cob surface, thus damage is not so bulky.

The ∼Cry1Ab toxin content (corrected with toxin/protoxin cross-reactivity [11,12]) was found to be 826 ng (s.d. = 237); 1280 ng (s.d. = 150) and 2075 ng (s.d. = 1287) ∼CryAb toxin/g fresh mass in corn silk; husk leaves and young cob, respectively. The high standard deviation in the cob is most likely due to this plant part being a mixture of different tissues with variable amounts of ∼Cry1Ab toxin.

Apical infection by *H. armigera* larvae—the most frequent damage type (Table 4)—results in the loss of only 10–15% of the entire cob (Table 6). Infection and damage on the base and the middle of the cobs might cause more damage.

### 3.3. Effect of MON 810 on *Helicoverpa armigera* under Field Conditions

MON 810 maize shows a high efficacy against *H. armigera* and *O. nubilalis* larval infection. None of the *O. nubilalis* larvae survived in stems, but some in maize ears (Table 7). There are some survivors in the case of *H. armigera* in maize ears. MON 810 drastically reduced *F. verticillioides* infection as well.

## 4. Discussion

### 4.1. Lepidoptera Larvae and *Fusarium verticillioides* Mycelia

It was apparent from the laboratory experiments that young larvae of *H. armigera* and *O. nubilalis* do not tolerate well *F. verticillioides* mycelia in their food (Table 1). In a laboratory experiment, *H. armigera* L1 could develop only until the third larval stadium (data not shown). *O. nubilalis* larvae could tolerate *F. verticillioides* mycelia much better. Nonetheless, *H. armigera* and *O. nubilalis* larvae feeding on *F. verticillioides* mycelia for a short period can spread fungal microconidia, which can survive the digestive channel of these insects. *F. verticillioides* was readily identified from the fecal pellets of both species. *H. armigera* larvae usually attempt to escape from moldy tunnels. During this period, the larva can transmit *F. verticillioides*. Evaluating co-occurrence of insect and fungal infection on the isogenic maize line, only 20–40% of the larva feeding on *F. verticillioides* infected seeds was found to transmit maize pink ear rot (Table 1). Similarly, some 20–30% of larval infection was found to be related to *F. verticillioides* infection in the field experiments (Table 2). In contrast, MON 810 maize, highly effective against the larvae, strongly reduced the *F. verticillioides* infection as well (Table 7). Incidental wounds on the top of maize ears [13] may play a more important role in this relationship than direct transmission. Insecticide (lambda-cyhalothrin) application against *O. nubilalis*, monoculture and effects of sowing date also indicate this effect [14–18]. In agreement with reported results [19,20], infestation through intact maize silk with *F. verticillioides* did not occur. Certain, but not all *Fusarium* mycotoxins were found to be related to *O. nubilalis* damage on *Bt*-plants (SYN-EV176 and MON 810 events) [21].

### 4.2. Effectivity of MON 810 Maize

MON 810 maize is advertised to be used against *O. nubilalis* larvae, which usually feed on the base of the leaves after hatching and subsequently make a tunnel into the stem (Table 4). MON 810 maize leaves produce the largest concentration and amount of the truncated toxin (*ca.* 10,000 ng ∼CryAb toxin/g fresh mass), while only a moderate concentration (*ca.* 1,500 ng ∼CryAb toxin/g fresh mass) is produced in the stem [11,12]. Different parts of the maize ear produce moderate (husk leaves, cobs) or low (maize silk) levels of ∼Cry1Ab toxin, therefore, are more suitable plant parts for the survival of those larvae which can feed on it. This is the probable reason why first instar larvae usually survive in the upper part of the maize ear feeding first on the maize silk. Similarly to reported results [22], no survival of *O. nubilalis* larvae was seen in the stem of MON 810 (Tables 1 and 7), but several survived in the lower part of the cob creating tunnels in the base of the husk leaves [23,24]. Larvae surviving in ∼Cry1Ab toxin containing maize ear with variable toxin content may become the source of Cry1-resistant strains in the future [25,26]. This applies especially to spots near the rescue zone, where intraspecific hybrid seeds with only one transgenic parental line and, consequently, lower Cry1Ab toxin production levels than the parental GMO trait (data not shown), are frequent.

### 4.3. Yield Loss is Caused by *Helicoverpa armigera, Ostrinia nubilalis* and *Fusarium verticillioides*

Although the most frequent apical damage of maize ear is very spectacular (Tables 4 and 5), the yield loss is rather moderate. High cob damage (40–50%) caused by *H. armigera* and/or *O. nubilalis* larvae is very rare in Hungary, and the yield loss is only 4–8% of the cob mass even in those infrequent cases (corresponding to 10–16% cob mass, Table 6). In reality, yield losses due to infected cob tops are even smaller, as seeds on the thinner top part of the cobs are often lost anyway during mechanic shelling of cobs. As the fungal infection occurs predominantly apically on the cobs, such loss of cob tops also reduces the average fusarotoxin content in the shelled kernels. This is the basic reason why Hungarian farmers practically do not use chemical insecticides against *H. armigera* and *O. nubilalis*, even though authorized preparations are available [27].

## 5. Conclusions

Laboratory experiments using third and fourth larval stages of *O. nubilalis* and *H. armigera* revealed that: (i) ∼Cry1Ab toxin distribution in MON 810 plants modifies survivorship of a single larva depending on where it attempts to enter the plant; (ii) *O. nubilalis* larvae prefer feeding in stems, while *H. armigera* larvae feed on corn ears only.

Field experiments with natural infestation indicated: (iii) *H. armigera* larvae tend to change feeding place in case of *F. verticillioides* infection, when they attempt to reach kernels via husk leaves; (iv) early infestation caused mostly apical maize ear damage; (v) only in 20–30% of cases was larval damage followed by a *F. verticillioides* infection; (vi) eventual yield loss is only about one-tenth of the corn ear apical infection; (vii) MON 810 maize effectively controls maize ear infection by *H. armigera* and *O. nubilalis*, but some larvae may survive leading to faster development of Cry1Ab-resistance in the future.

## Figures and Tables

**Table 1:** *Fusarium verticillioides* infestation transmitted by *Helicoverpa armigera* or *Ostrinia nubilalis* larva in maize ear isolator*.

**Maize**	**Treatment**	**Larval Damage[%]**		**Dead Larvae after Two Weeks [%]**	***Fusarium verticillioides* Mycelia after Two Weeks [%]**
		**Maize Ear**	**Stem**		
Isogenic	Untreated	-	-	-	0
	*Fusarium verticillioides* mycelia	-	-	-	0
	*Ostrinia nubilalis*	10	90	0	0
	*Ostrinia nubilalis + Fusarium verticillioides*	15	85	10	20
	*Helicoverpa armigera*	100	0	0	0
	*Helicoverpa armigera + Fusarium verticillioides*	100	0	17	42
MON 810	Untreated	-	-	-	0
	*Fusarium verticillioides* mycelia	-	-	-	0
	*Ostrinia nubilalis*	50	50	100	0
	*Ostrinia nubilalis + Fusarium verticillioides*	78	22	100	11
	*Helicoverpa armigera*	100	0	80	0
	*Helicoverpa armigera + Fusarium verticillioides*	100	0	71	0

* Notes: Larvae (repetitions 10–20) were individually separated and checked because of the solitary lifestyle and cannibalism.

**Table 2:** Percentage infestation rates by *Helicoverpa armigera*, *Ostrinia nubilalis* and *Fusarium verticillioides* on maize ears at three different plots at Julianna-major*.

**Hybrid**	**Maize Ear Investigated**	***Helicoverpa armigera***	***Ostrinia nubilalis***	**Larval Damage**	***Fusarium verticillioides***	***Fusarium* infection Related to Larval Damage**
Zamora	457	37.33 ± 4.78b	4.40 ± 0.88p	41.73 ± 4.07	8.13 ± 3.07x	19.76 ± 8.10v
DK-440 A	1339	21.32 ± 2.50a	22.84 ± 7.64q	44.16 ± 8.96	8.26 ± 1.62x	18.80 ± 2.41v
DK-440 B	578	43.34 ± 9.23b	2.83 ± 4.08p	46.17 ± 6.48	15.32 ± 4.24y	33.40 ± 9.64z

* Notes: Values followed by the same letter in a column are significantly not different from each other at 1% significance level (ANOVA, Tukey test). The distance between D-440 A and B plots was ∼1000 m; DK-440 A and Zamora ∼200 m; DK-440 B and Zamora ∼800 m.

**Table 3:** Position of *Ostrinia nubilalis* tunnels in same maize plants (Zamora).

**Tunnel in**	**Maize Plants Investigated**	**Infection Rate [%]**
Stem	581	73.03b
Maize ear	581	26.97a

Note: Values followed by different letters in a column are significantly different at 1% significance level (ANOVA, Tukey test).

**Table 4:** Position of *Ostrinia nubilalis* and *Helicoverpa armigera* tunnels in infected maize cobs (DK-440).

**Species**	**Tunnel in**	**Maize ears investigated**	**Damage rate [%]**
*Helicoverpa armigera*	base	286	9.98a
*Helicoverpa armigera*	top	286	90.02d
*Ostrinia nubilalis*	base	316	21.93b
*Ostrinia nubilalis*	top	316	78.07c

Note: Values followed by different letters in a column are significantly different at 1% significance level (ANOVA, Tukey test).

**Table 5:** Fungal infection related to larval damage (*Helicoverpa armigera* and *Ostrinia nubilalis*) of cobs (DK-440).

**Species**	**Damaged Cobs Investigated**	**Microbial Infection Rate [%]**
*Fusarium* spp.	602	16.82b
*Aspergillus* spp.	602	2.92a
*Penicillium* spp.	602	0.36a

Note: Values followed by the same letter in a column are significantly not different at 1% significance level (ANOVA, Tukey test).

**Table 6:** Cob loss by apical cob damage caused by *Helicoverpa armigera* larvae and *Fusarium verticillioides mycelia* (DK-440).

**Mass of Cob [g]**	**Mass of Cob Loss [%]**
Small cob [50–100]	15.82 ± 5.08 b
Medium cob [100–150]	12.84 ± 3.19ab
Big cob [150–200]	10.04 ± 2.63 a

* Note: Values followed by the same letter in a column are significantly not different from each other at 1% significance level (ANOVA, Tukey test).

**Table 7:** Efficacy of MON 810 maize (DK-440 BTY) on *Helicoverpa armigera*, *Ostrinia nubilalis* and *Fusarium verticillioides* infection.

**Species**	**Plant part**	**DK-440 [infection %]**	**DK-440 BTY [infection %]**	**Efficacy [%]**
*Helicoverpa armigera*	maize ear	37.33 ± 4.78 d	2.22 ± 2.07ab	94.05
*Ostrinia nubilalis*	maize ear	4.26 ± 0.79ab	0.39 ± 0.78 a	90.85
*Ostrinia nubilalis*	stem	16.55 ± 4.10 c	0.00 ± 0.00 a	100.00
*Fusarium verticillioides*	maize ear	7.43 ± 2.62 b	0.19 ± 0.39 a	97.44

* Note: Values followed by the same letter in this table are significantly not different at 1% significance level (ANOVA, Tukey test).
